# Prospective associations between media parenting practices and adolescent video game use

**DOI:** 10.1007/s12519-025-01009-y

**Published:** 2026-01-08

**Authors:** Jason M. Nagata, Derek Sportsman, Jennifer H. Wong, Sahana Nayak, Elizabeth J. Li, Kyle T. Ganson, Timothy Piatkowski, Jinbo He, Alexander Testa, Fiona C. Baker

**Affiliations:** 1 Division of Adolescent and Young Adult Medicine, Department of Pediatrics, University of California, San Francisco, 550 16th Street, 4th Floor, Box 0503, San Francisco, CA 94158 USA; 2 Factor-Inwentash Faculty of Social Work, University of Toronto, 246 Bloor St W, Toronto, ON M5S 1V4 Canada; 3 School of Applied Psychology and Griffith Centre for Mental Health, Griffith University, 176 Messines Ridge Road, Mt Gravatt, Brisbane, QLD 4122 Australia; 4 Centre for Health Services Research, The University of Queensland, Brisbane, Australia; 5 Department of Biosciences and Bioinformatics, School of Science, Xi’an Jiaotong-Liverpool University, Suzhou, 215123 China; 6 Department of Management, Policy and Community Health, University of Texas Health Science Center at Houston, 7000 Fannin St, Houston, TX 77030 USA; 7 Center for Health Sciences, SRI International, Menlo Park, 333 Ravenswood Ave, Menlo Park, CA 94025 USA

**Keywords:** Digital media, Parenting, Screens, Technology, Video games

## Abstract

**Background:**

Despite the rise of adolescent video gaming, evidence-based parenting guidelines and research on its specific behavioral impacts remain limited. This study evaluated whether media parenting practices are prospectively associated with video game use in adolescents 1 and 2 years later.

**Methods:**

We analyzed 7407 adolescents (51.6% male, age: 12.9 ± 0.6 years) from the Adolescent Brain Cognitive Development Study (year 3: 2019–2021 to year 5: 2021–2023). Multiple mixed-effects ordinal logistic regression and generalized linear models assessed the associations between parent media practices (screen time modeling, mealtime screen use, bedroom screen use, use to control behavior, monitoring and limiting) and video game behaviors (mature-rated games, problematic use and weekend video game time) 1 and 2 years later, adjusting for covariates.

**Results:**

Higher parental screen time modeling, mealtime screen use and bedroom screen use were associated with higher odds of playing mature-rated video games, whereas higher parental monitoring of screen time and limiting screen time were associated with lower odds of playing mature-rated video games and less total video game use 1 and 2 years later. Higher mealtime screen use, bedroom screen use and use of screens to control behavior were associated with greater total video game use 1 and 2 years later.

**Conclusions:**

This study demonstrates that certain media parenting practices can reduce adolescent video game use, while low parental involvement is linked to more problematic video game use behaviors. This study shows that parenting practices, including screen modeling, may influence adolescents’ video game behaviors.

**Graphical abstract:**

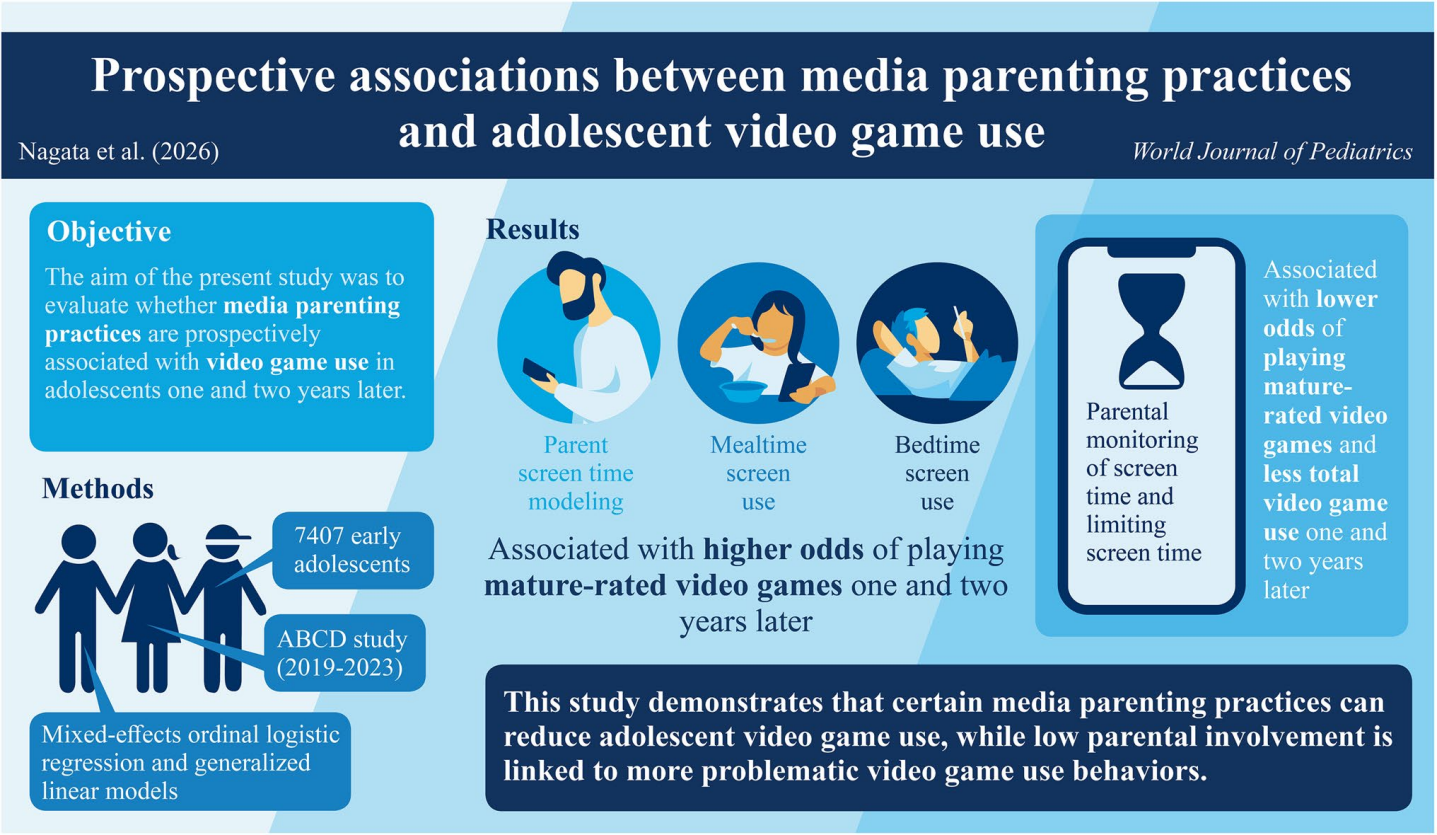

**Supplementary Information:**

The online version contains supplementary material available at 10.1007/s12519-025-01009-y.

## Introduction

With screen time becoming a common aspect of daily life, children aged 8–12 years spend an average of 5.5 hours per day on screens, while adolescents aged 13–18 years average 8.5 hours daily [1]. Although screen time is typically quantified in hours and minutes per day, such metrics overlook the context, content or potentially problematic aspects of use [2, 3]. Digital media use has been associated with elevated likelihoods of insomnia, depression and anxiety [4]. However, evidence-driven parenting guidelines for adolescent screen use remain limited [5–7].

As of 2024, about 85% of U.S. teenagers report playing video games, with 41% playing daily [8]. While both boys and girls commonly play video games, boys are more likely to identify as gamers (62% vs. 17%) and play daily (61% vs. 22%) [8]. While often associated with negative outcomes, young adult video gamers have been found to be better at tasks involving visual attention, working memory and task switching compared to non-gamers [9]. Importantly, not all adolescents who engage in frequent gaming experience negative consequences. Nonetheless, gaming for more than two hours a day has been shown to have associations with elevated rates of depressive symptoms, obsessive–compulsive behaviors, suicidal ideation and behavioral issues in children and adolescent populations [10]. Problematic video gaming is another rising issue and is characterized by persistent and maladaptive patterns of video game use that can lead to impairments in daily functioning and psychosocial well-being [11]. Problematic video gaming affects an estimated 1%–3% of adolescents and adults worldwide, with its underlying neural activity closely mirroring that found in substance use disorders [11, 12].

Beyond general gaming, game content is an important consideration. Mature-rated media, which include adult language, violence, sexual content, and substance use, are increasingly accessible to adolescents [13]. More than half of adolescents (56%) report exposure to mature-rated media by age 11 [13]. Notably, exposure to mature video games has been associated with high-risk behaviors, including substance use and early smoking initiation [13, 14].

A balanced parenting strategy that blends knowledge and understanding of gaming with firm yet supportive boundaries has been shown to be most effective for promoting healthy video game use among teens [15]. According to social learning theory (SLT), media parenting practices influence adolescents’ screen behaviors, as adolescents observe and imitate their parents’ habits [33]. While existing literature has explored how media-related parenting behaviors relate to adolescent screen time more broadly [5, 16, 17], few studies have focused specifically on video game use. Although one prior study focused on how parental mediation and social skills shape escapist motivations through online video gaming, it relied on a cross-sectional sample of 452 adolescents [18]. A larger cross-sectional study found that adolescents who perceived their parents as controlling exhibited higher levels of problematic gaming compared to those who perceived their parents as supportive and uninvolved [19]. There is, therefore, a critical need for comprehensive and longitudinal research on the association between parental media practices and adolescent video game use over time. The present study aims to address this gap by assessing the prospective associations between media parenting practices (e.g., parental screen time modeling, or the extent to which parents engage in screen use in their child’s presence, mealtime screen use, bedroom screen use, screen use to control behavior, parental screen time monitoring and limiting screen time) and video game behaviors 1 and 2 years later amongst a diverse, national sample of early adolescents in the U.S. We hypothesized:

1. Screen time modeling, mealtime and bedroom screen use, and the use of screens to control behavior would be associated with playing more mature-rated video games, greater problematic video game use, and greater time spent playing video games.
2. Monitoring and limiting screen time would be associated with less mature-rated video game use, problematic video game use, and time spent playing video games.
3. (Exploratory): the strength and direction of the associations in hypotheses one and two may differ by sex, such that males and females could be differentially affected by specific media parenting practices (e.g., screen time modeling or parental limits) in relation to mature-rated video game use, problematic gaming, and video game time.

## Methods

### Study population

We analyzed data from year 3 (2019–2021, 11–13 years old) to year 5 (2021–2023, 14–16 years old) of the Adolescent Brain Cognitive Development (ABCD) study, a longitudinal cohort study of adolescents in the U.S. At baseline (2016–2018), 11,875 adolescents were recruited from 21 study sites across the U.S. Recruitment and sampling primarily occurred through educational institutions, with attention to factors such as sex, race and ethnicity and socioeconomic status to reduce selection bias. Further details about the structure and recruitment of study participants are described elsewhere [20].

This study utilized data from the ABCD 6.0 release. Participants with missing socio-demographic (*n* = 442), media parenting practices (*n* = 1971) and video game use (*n* = 2142) data were excluded, resulting in a final sample size of 7407 adolescents. Participants reporting no video game use were retained in the study. Compared to the included sample, excluded participants were more likely to be from racial or ethnic minorities, households with lower income and to have parents with a high school education or less (Supplementary Material 1). Written informed consent was obtained from parents/caregivers and assent was obtained from participants. Institutional review board approval was granted by the University of California, San Diego and each study site.

### Media parenting practices

Media parenting practices were assessed at year 3 (2019–2021). Parents completed a 14-item self-report questionnaire to assess their typical daily screen time practices with their children [21]. The questionnaire was derived from a previously validated measure of media parenting practices [22]. Responses were aggregated into six categories: (1) parental screen time modeling (e.g., “I try to limit how much I use a screen-based device when I am with my child” and “When I am with my child, I use a screen-based device”); (2) mealtime screen use (e.g., “Our family often watches a screen during meals”); (3) bedroom screen use (e.g., “My child falls asleep while using a screen-based device”); (4) use of screens to control behavior (e.g., “I offer screen time to my child as a reward for good behavior”); (5) parental monitoring of screen time (e.g., “I keep track of my child’s screen time during the week”); and (6) parental limiting of screen time (e.g., “I limit my child’s screen time during the week”) [22]. Responses were rated on a four-point Likert-type scale from 1 (strongly disagree) to 4 (strongly agree). Scores for each of the six categories were calculated by averaging participants’ responses to the items within that specific category. For the parental screen time modeling category, the item “I try to limit how much I use a screen-based device when I am with my child” was reverse-coded to maintain consistency in directionality with the question, “When I am with my child, I use a screen-based device” prior to averaging the responses. The McDonald’s ω, a reliability coefficient indicating how consistently items measure the same underlying construct, was 0.64, suggesting acceptable internal consistency [23].

### Mature-rated video games

Mature-rated video game usage was assessed at years 4 (2020–2022) and 5 (2021–2023) using the ABCD Youth Screen Time Survey. Adolescents self-reported their frequency of playing mature-rated video games through the question, “How often do you play mature-rated video games (e.g., Call of Duty, Grand Theft Auto and Assassin’s Creed)?”. Responses were on a scale from 0 (never) to 3 (all the time). Participants who did not report any video game use were imputed as “never” on the above item.

### Problematic video game use

Adolescent-reported problematic video game use was assessed at years 4 (2020–2022) and 5 (2021–2023) using the six-item Video Game Addiction Questionnaire (VGAQ). The questions were adapted from the Bergen Facebook Addiction Scale [24], which is a unidimensional questionnaire originally designed to assess Facebook addiction. However, previous studies have extended its use to evaluate broader video game and social media addiction among high school and college students [25, 26]. Example items include: “I play video games so much that it has had a bad effect on my schoolwork or job” and “I feel the need to play video games more and more”. Responses were recorded on a Likert-type scale ranging from 1 (never) to 6 (very often). Participants who did not report any video game use were imputed as “never” on the above items. The McDonald’s ω was 0.87, indicating strong internal consistency. Currently, there are no established guidelines for defining a cut-off score for problematic gaming using this questionnaire [27]. However, higher scores may reflect greater symptoms of problematic gaming.

### Adolescent-reported video game use

Video game time was assessed at years 4 (2020–2022) and 5 (2021–2023) using the ABCD Youth Screen Time Survey. Participants reported the number of hours per day playing single-player video games and multiplayer video games on weekend days [28]. Starting in year 5, only weekend video game use was collected; therefore, analyses focused on weekends [29]. Single-player and multiplayer video game use were summed to create a total weekend game time variable as a continuous outcome (hours/day). Video game time was winsorized at 20 hours/day (≥ 98th percentile). Winsorization reduces the influence of extreme outliers by capping them at specific percentiles and replacing outliers with the values at the percentiles.

### Covariates

Several year 3 covariates were included in the analysis to account for potential socio-demographic and contextual factors that may be associated with media parenting practices and video games. Covariates included age, sex (female or male), race and ethnicity (Asian, Black, Hispanic/Latino, Native American, White or other), annual household income (< $25,000, $25,000–$49,999, $50,000–$74,999, $75,000–$99,999, $100,000–$199,999 and ≥ $200,000), highest parental education level (high school or less compared with college or more), the year 3 value of the same video game outcome assessed at years 4 and 5, study year and study site. Missing age, income and parental education data were imputed using the last observation carried forward method.

### Statistical analyses

Analyses were conducted in Stata 18.0 (StataCorp, College Station, TX). Multiple mixed-effects ordinal logistic regression models with robust standard errors estimated the association between media parenting practices and the frequency of playing mature-rated video games. Multiple mixed-effects generalized linear models with robust standard errors estimated the association between media parenting practices and problematic video game use and video game time. A random intercept was included for each participant to account for repeated measures. All models adjusted for the aforementioned covariates. We tested for interaction by sex and presented results stratified by sex if there was evidence of interaction (*P* for interaction < 0.05). ABCD sampling weights were applied to all analyses to approximate the socio-demographic characteristics of the U.S. population represented by the American Community Survey from the U.S. Census [30]. Testing was two-sided, and *P* < 0.05 was considered statistically significant.

## Results

In the sample of 7407 adolescents, the average age at year 3 was 12.9 years [standard deviation (SD) = 0.6], 51.6% were male, and 43.3% were from racial/ethnic minorities (Table 1). Table 2 shows the prospective associations between parent media practices and the frequency of playing mature-rated video games, problematic video game use and video game time. Greater parental screen time modeling, such as a parent’s own use of a screen-based device in the presence of their child, was associated with higher odds of playing mature-rated video games [adjusted odds ratio (AOR) 1.08, 95% confidence interval (CI) 1.02, 1.14]. Higher mealtime screen use was associated with higher odds of playing mature-rated video games (AOR 1.10, 95% CI 1.06, 1.14). Higher bedroom screen use was associated with higher odds of playing mature-rated video games (AOR 1.08, 95% CI 1.04, 1.13). More parental monitoring was associated with lower odds of reporting mature-rated video games (AOR 0.94, 95% CI 0.91, 0.97). Greater screen time limits were associated with lower odds of reporting mature-rated video games (AOR 0.90, 95% CI 0.86, 0.95).

**Table 1:** Participant characteristics in the Adolescent Brain Cognitive Development (ABCD) study (*N* = 7407)

Characteristics	Values
Age (y) (year 3)	12.9 (0.6)
Sex	
Female	48.4
Male	51.6
Race and ethnicity	
Asian	5.7
Black	14.9
Latino/Hispanic	18.5
Native American	2.9
Other	1.2
White	56.7
Household income (year 3)	
$24,999 or less	13.3
$25,000 to $49,999	15.4
$50,000 to $74,999	15.3
$75,000 to $99,999	15.2
$100,000 to $199,999	29.7
$200,000 or greater	11.2
Parent's highest education (year 3)	
High school education or less	13.4
College education or more	86.6
Media parenting practices (year 3)^a^	
Parental screen time modeling	2.3 (0.6)
Mealtime screen use	1.9 (0.9)
Bedroom screen use	1.8 (0.9)
Use of screens to control behavior	2.6 (0.9)
Parental monitoring of screen time	2.7 (1.0)
Limiting screen time	3.1 (0.7)
Frequency of playing mature rated video games (year 3)	
Never	56.0
Once in a while	24.1
Regularly	13.5
All the time	6.4
Frequency of playing mature rated video games (year 4)	
Never	52.2
Once in a while	24.7
Regularly	14.9
All the time	8.2
Frequency of playing mature rated video games (year 5)	
Never	52.5
Once in a while	24.5
Regularly	14.4
All the time	8.6
Problematic video game use (year 3)^b^	2.0 (1.1)
Problematic video game use (year 4)^b^	1.9 (1.0)
Problematic video game use (year 5)^b^	1.9 (1.1)
Total weekend video game time (year 3)	3.4 (4.1)
Total weekend video game time (year 4)	4.1 (4.6)
Total weekend video game time (year 5)	3.5 (4.3)

Values are presented as mean (SD) or %. ABCD study sampling weights were applied based on the American Community Survey from the U.S. Census. *SD* standard deviation

^a^Responses were rated on a four-point Likert-type scale from 1 (strongly disagree) to 4 (strongly agree)

^b^Responses were rated on a six-point Likert-type scale from 1 (strongly disagree) to 6 (strongly agree)

**Table 2:** Associations between media parenting practices (year 3) and video games (years 4 and 5) in the Adolescent Brain Cognitive Development (ABCD) study (*N* = 7407)

Media parenting practice categories	Frequency of mature video games		Problematic video game use		Total video game time (h/d)	
	AOR (95% CI)	*P*	*B* (95% CI)	*P*	*B* (95% CI)	*P*
Parental screen time modeling	**1.08 (1.02, 1.14)**	**0.005**	0.02 (− 0.0001, 0.04)	0.051	0.05 (− 0.03, 0.14)	0.242
Mealtime screen use	**1.10 (1.06, 1.14)**	**< 0.001**	**0.02 (0.01, 0.04)**	**0.001**	**0.17 (0.11, 0.23)**	**< 0.001**
Bedroom screen use	**1.08 (1.04, 1.13)**	**< 0.001**	− 0.01 (− 0.02, 0.01)	0.482	**0.08 (0.01, 0.15)**	**0.021**
Use of screens to control behavior	1.03 (0.99, 1.07)	0.143	**0.03 (0.02, 0.05)**	**< 0.001**	**0.17 (0.10, 0.23)**	**< 0.001**
Parental monitoring of screen time	**0.94 (0.91, 0.97)**	**< 0.001**	− 0.01 (− 0.02, 0.003)	0.146	− **0.06 (**− **0.12,** − **0.002)**	**0.044**
Limiting screen time	**0.90 (0.86, 0.95)**	**< 0.001**	− 0.01 (− 0.02, 0.01)	0.536	− **0.14 (**− **0.22,** − **0.06)**	**0.001**

Bold indicates *P* < 0.05. Models represent the abbreviated output from the mixed-effects ordinal logistic regression model or mixed-effects generalized linear models with adjustment for year 3 age, sex, race and ethnicity, household income, highest parental education, respective video game variable, study year, and study site. *AOR* adjusted odds ratio from mixed-effects ordinal logistic regression model, *B* beta from mixed-effects generalized linear model, *CI* confidence interval

Higher mealtime screen use was associated with greater problematic video game use [beta (*B*) 0.02, 95% CI 0.01, 0.04]. Beta (*B*) represents the estimated change in the outcome variable associated with a one-unit increase in the predictor; in this case a *B* of 0.02 indicates that higher mealtime screen use is positively associated with slightly greater problematic video game use. Greater use of screens to control behavior was associated with greater problematic video game use (*B* 0.03, 95% CI 0.02, 0.05).

Higher mealtime screen use was associated with greater time spent playing video games (*B* 0.17, 95% CI 0.11, 0.23). Higher bedroom screen use was associated with greater time spent on video games (*B* 0.08, 95% CI 0.01, 0.15). Greater use of screens to control behavior was associated with greater time spent playing video games (*B* 0.17, 95% CI 0.10, 0.23). More parental monitoring was associated with less time spent on video games (*B* − 0.06, 95% CI − 0.12, − 0.002). Greater screen time limits were associated with less time spent on video games (*B* − 0.14, 95% CI − 0.22, − 0.06). Group-based trajectory model fit statistics are reported in Supplementary Material 3.

We observed significant sex interactions between parent media practices and video game behaviors (Supplementary Material 2). Greater screen time modeling was associated with higher odds of playing mature-rated video games among males (AOR 1.12, 95% CI 1.05, 1.21). Greater parental monitoring was associated with lower problematic video game use among males (*B* − 0.03, 95% CI − 0.05, − 0.01). Greater mealtime screen use was associated with more time spent playing video games among females (*B* 0.14, 95% CI 0.06, 0.21) and males (*B* 0.21, 95% CI 0.11, 0.30).

## Discussion

The aim of this study was to examine whether media parenting practices predicted adolescent video game use 1 and 2 years later. In a sample of over 7400 adolescents, parental screen time modeling, screen use during meals or in bedrooms, and using screens to control behavior were linked to greater gaming, including problematic and mature-rated game play. Parental monitoring and limits on screen time were linked to lower gaming engagement. Although statistically significant, these associations were small in magnitude.

### Parental screen time modeling

Greater parental screen time modeling, such as using screens in the presence of adolescents, was prospectively associated with higher odds of mature video game use. These findings align with prior cross-sectional studies which found that higher parental screen time modeling was associated with a higher likelihood of playing mature video games [31]. This study builds on previous research by establishing a temporal relationship, which demonstrates that parental screen time modeling has a prospective association with higher odds of mature video game use 1 and 2 years later.

These prospective associations can potentially be explained by two mechanisms: first, with more time spent on their own devices, parents may enforce fewer limitations and rules on their children’s screen use [32]; this may contribute to more frequent video game use. Another possible explanation is through the SLT [33]; as parents model screen use, adolescents reflect this behavior, resulting in subsequently higher screen and video game use. A cross-sectional study identified a link between parental phubbing, defined as parents’ use of mobile devices instead of engaging with their children, and greater adolescent mobile video game use [34]. In this context, adolescents may turn to video gaming, which may include mature content, as a response to the lack of parental attention [34]. For these reasons, modification of parental screen time habits is a promising target intervention for reducing adolescent video game use.

### Mealtime and bedroom screen use

Greater mealtime and bedroom screen use were prospectively associated with a higher frequency of mature video game use and greater overall time spent playing video games. Mealtime screen use showed associations with higher problematic video game use. These findings align with prior cross-sectional research suggesting that there are associations between mealtime screen use, bedroom screen use and more time spent playing video games [35–38]. These findings may be attributed to parental phubbing during mealtime and bedroom screen use, and may be explained by the intergenerational transmission theory, which suggests such behaviors can be passed down to children, increasing the risk of problematic gaming, including mature-rated video games [34]. Without mealtime and bedroom screen use limits, families likely have very few protected times in the household when screen use is limited. Thus, it is unsurprising that screen use during mealtime and bedroom time was associated with measures of greater video game use.

### Use of screens to control behavior

The use of screens as a consequence or reward to control adolescent behavior was prospectively associated with higher problematic video game use and greater time spent playing video games. These findings are consistent with previous cross-sectional literature, which demonstrated associations between parental use of screens to control behavior and greater problematic video gaming and screen time, which included television, cell phones, iPads or tablets and video games [5, 21]. Interestingly, the association between control-based media parenting practices and higher problematic video game use was consistent in settings of both punishment and reward. This finding may be explained by the pursuit of autonomy in early adolescence; perceived intrusiveness from parental figures may contribute to resistance toward media-related regulation [39–42]. Furthermore, the framing of screens as a reward may increase adolescents’ desire to use them, leading to greater video game use overall.

### Parental monitoring of screen time

Higher parental monitoring of screen time was prospectively associated with lower odds of mature video game use and lower video game time. The impact of parental monitoring on video game use is yet to be confirmed in current literature; some cross-sectional studies found no significant associations between parental monitoring of screen time and adolescent video game use [5, 38], while another cross-sectional study found that parental monitoring was associated with lower mature video game use [31]. Regardless, our study suggests that parental monitoring of screen time could be an effective intervention to reduce adolescent video game use.

### Limiting screen time

Greater parental limiting of adolescent screen time was prospectively associated with lower odds of mature video game use and lower video game time. Similarly, past cross-sectional studies demonstrated that screen time limits were associated with lower total screen time [5, 35], lower problematic screen use [5] and a lower likelihood of playing mature video games [31]. This prospective cohort study further contributes to this body of research, leveraging temporal relationships to demonstrate an association between the establishment of parental screen time limits and adolescent video game use 1 and 2 years later. These findings suggest that parental rules regarding video game use can potentially impact adolescent behavior and thus may be an effective and sustainable intervention to reduce video game use throughout early to late adolescence.

### Sex differences in parent media practices

Our findings suggest that the influence of media parenting practices on adolescent gaming behaviors may differ by sex. Greater parental screen time modeling was associated with playing more mature-rated video games among males. In addition, greater parental monitoring was linked to lower problematic video game use among males. Moreover, greater mealtime screen use was associated with more time spent playing video games among both males and females. Although statistically significant interactions by sex were observed and the direction of effects was consistent with the main analyses, the effect sizes were small. These findings diverge from a previous ABCD study investigating media parenting practices and adolescent mature-rated video games and R-rated movies [31]. In the current analysis, only screen time modeling showed significant sex-interactions on mature video game play, whereas the previous study reported significant sex-interactions for bedroom screen use, monitoring, and limiting on mature video game play. This indicates the need for further investigation into the sex-specific effects of media parenting practices on video game behaviors. Future studies should also examine the interaction between parental and adolescent biological sex on gaming behaviors.

### Strengths and limitations

Strengths of this study include the use of data from a large, nationally representative prospective cohort with 1- and 2-year follow-up, enhancing the generalizability of our findings. However, exclusions due to missing data may limit generalizability to lower-income and minority populations. Limitations include the use of self-reported data, which potentially introduces recall bias or social desirability bias. The media parenting practice questionnaire had low, but acceptable, internal consistency (McDonald’s ω = 0.64), suggesting the need for refinement in future research. Media parenting practice items did not differentiate between screen content types, potentially impacting the observed associations. However, preliminary analyses between media parenting practices and adolescent video game time support our approach, justifying further analysis of mature-rated and problematic use despite this limitation. In addition, video game time was measured only at weekends, which may limit generalizability to overall adolescent gaming. Some findings had a small effect size with confidence intervals approaching the null, suggesting the possibility of type I error and warranting cautious interpretation. Though this study adjusted for covariates such as socio-demographic factors and year 3 video game outcomes, unknown confounders could exist, such as frequency of parental video gaming and parental biological sex. Future studies could account for these potential confounders.

In conclusion, video gaming has become increasingly popular amongst the younger population, with over 90% of children over the age of 2 years playing video games, and three-quarters of households in the U.S. owning video game consoles [43]. Clarifying and understanding the role of media parenting practices as they relate to video game use in adolescents is critical for informing future evidence-based clinical and policy guidelines. Our prospective findings examining the associations between media parenting practices and video game behaviors may also contribute to a more nuanced understanding of the ways in which parental strategies can shape adolescent screen time habits. Although the study cannot establish causality, the observed associations highlight potential targets for family-based media management. Future studies should implement interventions to moderate parental screen time and assess their strengths in reducing adolescent video game use.

## Supplementary Information

Below is the link to the electronic supplementary material.

Supplementary file1 (DOCX 217 KB)

## Data Availability

Data used in the preparation of this article were obtained from the ABCD Study (https://abcdstudy.org), held in the NIH Brain Development Cohorts (NBDC) Portal.

## References

[1] Nagata JM, Al-Shoaibi AAA, Leong AW, Zamora G, Testa A, Ganson KT, et al. Screen time and mental health: a prospective analysis of the Adolescent Brain Cognitive Development (ABCD) Study. BMC Public Health. 2024;24:2686. 39370520 10.1186/s12889-024-20102-x PMC11457456

[2] Yu H, Xu C, Lu J, Li Q, Li Q, Zhou K, et al. Associations between screen time and emotional and behavioral problems among children and adolescents in US, National Health Interview Survey (NHIS), 2022. J Affect Disord. 2025;379:159–67. 40081579 10.1016/j.jad.2025.03.030

[3] Santos RMS, Mendes CG, Sen Bressani GY, de Alcantara Ventura S, de Almeida Nogueira YJ, de Miranda DM, et al. The associations between screen time and mental health in adolescents: a systematic review. BMC Psychol. 2023;11:127. 37081557 10.1186/s40359-023-01166-7 PMC10117262

[4] Muppalla SK, Vuppalapati S, Reddy Pulliahgaru A, Sreenivasulu H. Effects of excessive screen time on child development: an updated review and strategies for management. Cureus. 2023;15:e40608. 37476119 10.7759/cureus.40608 PMC10353947

[5] Nagata JM, Paul A, Yen F, Smith-Russack Z, Shao IY, Al-Shoaibi AAA, et al. Associations between media parenting practices and early adolescent screen use. Pediatr Res. 2025;97:403–10. 38834780 10.1038/s41390-024-03243-y PMC11626836

[6] Veldhuis L, van Grieken A, Renders CM, Hirasing RA, Raat H. Parenting style, the home environment, and screen time of 5-year-old children; the ‘be active, eat right’ study. PLoS One. 2014;9:e88486. 24533092 10.1371/journal.pone.0088486 PMC3922818

[7] Geurts SM, Koning IM, Vossen HGM, van den Eijnden RJJM. Rules, role models or overall climate at home? Relative associations of different family aspects with adolescents’ problematic social media use. Compr Psychiatry. 2022;116:152318. 35537295 10.1016/j.comppsych.2022.152318

[8] Gottfried J, Sidoti O. Teens and video games today. Pew Research Center. 2024. https://www.pewresearch.org/internet/2024/05/09/teens-and-video-games-today/. Accessed 5 Aug 2025.

[9] Alho K, Moisala M, Salmela-Aro K. Effects of media multitasking and video gaming on cognitive functions and their neural bases in adolescents and young adults. Eur Psychol. 2022;27:131–40.

[10] Lager KS, Corso G. Game faces: how digital play affects the psychological landscape of youth. Cureus. 2025;17:e77497. 39958121 10.7759/cureus.77497 PMC11828491

[11] Marchica LA, Richard J, Nower L, Ivoska W, Derevensky JL. Problem video gaming in adolescents: an examination of the pathways model. Int Gambl Stud. 2022;22:282–99.

[12] Gros L, Debue N, Lete J, van de Leemput C. Video game addiction and emotional states: possible confusion between pleasure and happiness? Front Psychol. 2019;10:2894. 32047450 10.3389/fpsyg.2019.02894 PMC6996247

[13] Zhang L, Oshri A, Carvalho C, Uddin LQ, Geier C, Nagata JM, et al. Prospective associations between sleep, sensation-seeking, and mature screen usage in early adolescents: findings from the Adolescent Brain Cognitive Development study. Sleep. 2025;48:zsae234. 39390801 10.1093/sleep/zsae234 PMC12477119

[14] Shao R, Wang Y. The relation of violent video games to adolescent aggression: an examination of moderated mediation effect. Front Psychol. 2019;10:384. 30846962 10.3389/fpsyg.2019.00384 PMC6394371

[15] Meriläinen M. Crooked views and relaxed rules: how teenage boys experience parents’ handling of digital gaming. MaC. 2021;9:62–72.

[16] Swider-Cios E, Vermeij A, Sitskoorn MM. Young children and screen-based media: the impact on cognitive and socioemotional development and the importance of parental mediation. Cogn Dev. 2023;66:101319.

[17] Lafton T, Wilhelmsen JEB, Holmarsdottir HB. Parental mediation and children’s digital well-being in family life in Norway. J Child Media. 2024;18:198–215.

[18] Commodari E, Consiglio A, Cannata M, la Rosa VL. Influence of parental mediation and social skills on adolescents’ use of online video games for escapism: a cross-sectional study. J Res Adolesc. 2024;34:1668–78. 39438433 10.1111/jora.13034 PMC11606255

[19] Bradt L, Grosemans E, de Cock R, Dupont B, Vansteenkiste M, Soenens B. Does parents’ perceived style of setting limits to gaming matter? The interplay between profiles of parental mediation and BIS/BAS sensitivity in problematic gaming and online gambling. J Adolesc. 2024;96:580–97. 37968846 10.1002/jad.12271

[20] Garavan H, Bartsch H, Conway K, Decastro A, Goldstein RZ, Heeringa S, et al. Recruiting the ABCD sample: design considerations and procedures. Dev Cogn Neurosci. 2018;32:16–22. 29703560 10.1016/j.dcn.2018.04.004 PMC6314286

[21] Tang L, Darlington G, Ma DWL, Haines J, Guelph Family Health Study. Mothers’ and fathers’ media parenting practices associated with young children’s screen-time: a cross-sectional study. BMC Obes. 2018;5:37. 30524742 10.1186/s40608-018-0214-4 PMC6276169

[22] Larios SE, Ayala GX, Arredondo EM, Baquero B, Elder JP. Development and validation of a scale to measure Latino parenting strategies related to children’s obesigenic behaviors. The parenting strategies for eating and activity scale (PEAS). Appetite. 2009;52:166–72. 18845197 10.1016/j.appet.2008.09.011 PMC2659497

[23] Hayes AF, Coutts JJ. Use omega rather than cronbach’s alpha for estimating reliability. But…. Commun Methods Meas. 2020;14:1–24.

[24] Andreassen CS, Torsheim T, Brunborg GS, Pallesen S. Development of a facebook addiction scale. Psychol Rep. 2012;110:501–17. 22662404 10.2466/02.09.18.PR0.110.2.501-517

[25] Hou Y, Xiong D, Jiang T, Song L, Wang Q. Social media addiction: its impact, mediation, and intervention. Cyberpsychology. 2019;13:Article 4.

[26] Simsek A, Elciyar K, Kizilhan T. A comparative study on social media addiction of high school and university students. Contemp Educ Technol. 2019;10:106–19.

[27] Lopez DA, Foxe JJ, van Wijngaarden E, Thompson WK, Freedman EG. The longitudinal association between reward processing and symptoms of video game addiction in the Adolescent Brain Cognitive Development Study. J Behav Addict. 2024;13:1051–63. 39656219 10.1556/2006.2024.00068 PMC11737415

[28] Bagot KS, Matthews SA, Mason M, Squeglia LM, Fowler J, Gray K, et al. Current, future and potential use of mobile and wearable technologies and social media data in the ABCD study to increase understanding of contributors to child health. Dev Cogn Neurosci. 2018;32:121–9. 29636283 10.1016/j.dcn.2018.03.008 PMC6447367

[29] Guerrero MD, Barnes JD, Chaput JP, Tremblay MS. Screen time and problem behaviors in children: exploring the mediating role of sleep duration. Int J Behav Nutr Phys Act. 2019;16:105. 31727084 10.1186/s12966-019-0862-x PMC6854622

[30] Paulich KN, Ross JM, Lessem JM, Hewitt JK. Screen time and early adolescent mental health, academic, and social outcomes in 9- and 10- year old children: utilizing the Adolescent Brain Cognitive Development℠ (ABCD) study. PLoS One. 2021;16:e0256591. 34496002 10.1371/journal.pone.0256591 PMC8425530

[31] Nagata JM, Li K, Sui SS, Talebloo J, Otmar CD, Shao IY, et al. Associations between media parenting practices and early adolescent consumption of R-rated movies and mature-rated video games. BMC Pediatr. 2025;25:90. 39905395 10.1186/s12887-024-05367-w PMC11792743

[32] Wong RS, Tung KTS, Rao N, Leung C, Hui ANN, Tso WWY, et al. Parent technology use, parent-child interaction, child screen time, and child psychosocial problems among disadvantaged families. J Pediatr. 2020;226:258–65. 32629010 10.1016/j.jpeds.2020.07.006

[33] Bandura A. Social learning theory. Can J Sociol Cah Can Sociol. 2019;2:321.

[34] Liu J, Xie T, Mao Y. Parental phubbing behavior and adolescents’ online gaming time: the mediating role of electronic health literacy. Behav Sci. 2024;14:925. 39457798 10.3390/bs14100925 PMC11504069

[35] Ramirez ER, Norman GJ, Rosenberg DE, Kerr J, Saelens BE, Durant N, et al. Adolescent screen time and rules to limit screen time in the home. J Adolesc Health. 2011;48:379–85. 21402267 10.1016/j.jadohealth.2010.07.013 PMC3058142

[36] Jusienė R, Urbonas V, Laurinaitytė I, Rakickienė L, Breidokienė R, Kuzminskaitė M, et al. Screen use during meals among young children: exploration of associated variables. Medicina. 2019;55:688. 31615125 10.3390/medicina55100688 PMC6843261

[37] Hale L, Kirschen GW, LeBourgeois MK, Gradisar M, Garrison MM, Montgomery-Downs H, et al. Youth screen media habits and sleep: sleep-friendly screen behavior recommendations for clinicians, educators, and parents. Child Adolesc Psychiatr Clin N Am. 2018;27:229–45. 29502749 10.1016/j.chc.2017.11.014 PMC5839336

[38] Smith LJ, Gradisar M, King DL. Parental influences on adolescent video game play: a study of accessibility, rules, limit setting, monitoring, and cybersafety. Cyberpsychol Behav Soc Netw. 2015;18:273–9. 25965861 10.1089/cyber.2014.0611

[39] De Lepeleere S, De Bourdeaudhuij I, Cardon G, Verloigne M. Do specific parenting practices and related parental self-efficacy associate with physical activity and screen time among primary schoolchildren? A cross-sectional study in Belgium. BMJ Open. 2015;5:e007209. 26346871 10.1136/bmjopen-2014-007209 PMC4563237

[40] Sanders W, Parent J, Forehand R, Sullivan AD, Jones DJ. Parental perceptions of technology and technology-focused parenting: associations with youth screen time. J Appl Dev Psychol. 2016;44:28–38. 27795603 10.1016/j.appdev.2016.02.005 PMC5082753

[41] Geurts SM, Koning IM, Vossen H, van den Eijnden RJJM. A qualitative study on children’s digital media use and parents’ self-interest. J Child Fam Stud. 2022;31:2015–26. 34580571 10.1007/s10826-021-02074-3 PMC8458790

[42] Poulain T, Meigen C, Kiess W, Vogel M. Media regulation strategies in parents of 4- to 16-year-old children and adolescents: a cross-sectional study. BMC Public Health. 2023;23:371. 36810002 10.1186/s12889-023-15221-w PMC9942333

[43] Alanko D. The health effects of video games in children and adolescents. Pediatr Rev. 2023;44:23–32. 36587018 10.1542/pir.2022-005666

