# Supplementary material for: Prospective associations between media parenting practices and adolescent video game use
Source: World J Pediatr. 2026 Jan 8;22(2):225–33. doi: 10.1007/s12519-025-01009-y (PMC12871385; doi:10.1007/s12519-025-01009-y)

| Supplementary Material 1. Comparison of participants included vs excluded in the Adolescent Brain Cognitive Development (ABCD) Study (*N* = 7407) | | | |
| --- | --- | --- | --- |
| Sociodemographic characteristics | Included | Excluded | *P* |
|  | (*n* = 7407) | (*n* = 4555) |  |
| Age | 12.9 (0.6) | 13.0 (0.7) | <0.001 |
| Sex |  |  |  |
| Female | 48.4% | 49.5% | 0.248 |
| Male | 51.6% | 50.5% |  |
| Race and ethnicity |  |  |  |
| Asian | 5.7% | 5.3% | <0.001 |
| Black | 14.9% | 21.2% |  |
| Latino/Hispanic | 18.5% | 22.6% |  |
| Native American | 2.9% | 3.6% |  |
| Other | 1.2% | 1.8% |  |
| White | 56.7% | 45.6% |  |
| Household income |  |  |  |
| $24,999 or less | 13.3% | 21,3% | <0.001 |
| $25,000 to $49,999 | 15.4% | 20.1% |  |
| $50,000 to $74,999 | 15.3% | 14.5% |  |
| $75,000 to $99,999 | 15.2% | 11.9% |  |
| $100,000 to $199,999 | 29.7% | 24.1% |  |
| $200,000 or greater | 11.2% | 8.1% |  |
| Parents' highest education |  |  |  |
| High school education or less | 13.4% | 22.7% | <0.001 |
| College education or more | 86.6% | 77.3% |  |

| Supplementary Material 2. Associations between media parenting practices and video games in the Adolescent Brain Cognitive Development (ABCD) Study (*N* = 7407), stratified by sex^a^ | | | | | | | | | | | | | | | |
| --- | --- | --- | --- | --- | --- | --- | --- | --- | --- | --- | --- | --- | --- | --- | --- |
|  | Frequency of Mature Video Games | | | | | Problematic Video Game Use | | | | | Total Video Game Time (h/d) | | | | |
|  | Female (*N* = 3539) | | Male (*N* = 3868) | |  | Female (*N* = 3539) | | Male (*N* = 3868) | |  | Female (*N* = 3539) | | Male (*N* = 3868) | |  |
| Media parenting practice categories | *AOR* (95% CI) | *P* | *AOR* (95% CI) | *P* | *P* for interaction^b^ | *B* (95% CI) | *P* | *B* (95% CI) | *P* | *P* for interaction^b^ | *B* (95% CI) | *P* | *B* (95% CI) | *P* | *P* for interaction^b^ |
| Parental screen time modeling | 1.06 (0.96, 1.17) | 0.263 | **1.12 (1.05, 1.21)** | **0.002** | **0.049** | 0.01 (-0.02, 0.04) | 0.465 | **0.03 (0.003, 0.06)** | **0.031** | 0.293 | **0.13 (0.02, 0.24)** | **0.023** | -0.01 (-0.14, 0.12) | 0.907 | 0.165 |
| Mealtime screen use | **1.09 (1.02, 1.16)** | **0.007** | **1.11 (1.06, 1.16)** | **< 0.001** | 0.149 | **0.02 (0.002, 0.04)** | **0.030** | **0.03 (0.01, 0.05)** | **0.007** | 0.370 | **0.14 (0.06, 0.21)** | **< 0.001** | **0.21 (0.11, 0.30)** | **< 0.001** | **0.018** |
| Bedroom screen use | **1.11 (1.04, 1.20)** | **0.003** | **1.08 (1.02, 1.14)** | **0.008** | 0.589 | -0.01 (-0.02, 0.01) | 0.536 | -0.004 (-0.03, 0.02) | 0.725 | 0.767 | 0.07 (-0.002, 0.15) | 0.058 | 0.09 (-0.02, 0.20) | 0.119 | 0.126 |
| Use of screens to control behavior | 0.99 (0.92, 1.07) | 0.841 | **1.06 (1.00, 1.11)** | **0.042** | 0.058 | **0.03 (0.01, 0.05)** | **0.002** | **0.03 (0.01, 0.05)** | **0.003** | 0.904 | **0.16 (0.08, 0.23)** | **< 0.001** | **0.19 (0.09, 0.28)** | **< 0.001** | 0.367 |
| Parental monitoring of screen time | **0.89 (0.84, 0.95)** | **< 0.001** | 0.96 (0.92, 1.01) | 0.097 | 0.701 | 0.01 (-0.01, 0.02) | 0.369 | **-0.03 (-0.05, -0.01)** | **0.011** | **0.008** | -0.03 (-0.10, 0.03) | 0.334 | -0.09 (-0.18, 0.01) | 0.073 | 0.231 |
| Limiting screen time | **0.83 (0.77, 0.91)** | **< 0.001** | 0.93 (0.87, 1.00) | 0.051 | 0.531 | 0.01 (-0.01, 0.04) | 0.198 | -0.03 (-0.06, 0.001) | 0.061 | **0.021** | **-0.10 (-0.20, -0.01)** | **0.035** | **-0.18 (-0.30, -0.05)** | **0.006** | 0.093 |
| ^a^ **Bold** indicates *P* < 0.05. Models represent the abbreviated output from the mixed-effects ordinal logistic regression model or mixed-effects generalized linear models with adjustment for Year 3 age, sex, race and ethnicity, household income, highest parental education, respective video game variable, study year, and study site. *AOR* adjusted odds ratio from mixed-effects ordinal logistic regression model, *B* coefficient from mixed-effects generalized linear model, *CI* confidence interval. | | | | | | | | | | | | | | | |
| ^b^ P-value for the media parenting practice*sex interaction term coefficient | | | | | | | | | | | | | | | |

| Supplementary Material 3. Model fit statistics of each mixed-effects ordinal logistic regression model and mixed-effects generalized linear model. | | | | | | | | | |
| --- | --- | --- | --- | --- | --- | --- | --- | --- | --- |
|  | Frequency of Mature Video Games | | | Problematic Video Game Use | | | Total Video Game Time (h/d) | | |
| Media parenting practice categories | *AIC* | *ICC* | *Random intercept variance* | *AIC* | *ICC* | *Random intercept variance* | *AIC* | *ICC* | *Random intercept variance* |
| Parental screen time modeling | 25,800,000 | 0.04 | 0.14 | 32,300,000 | 0.14 | 0.07 | 76,400,000 | 0.08 | 0.82 |
| Mealtime screen use | 25,700,000 | 0.04 | 0.13 | 32,300,000 | 0.14 | 0.07 | 76,300,000 | 0.08 | 0.80 |
| Bedroom screen use | 25,800,000 | 0.04 | 0.13 | 32,300,000 | 0.14 | 0.07 | 76,400,000 | 0.08 | 0.81 |
| Use of screens to control behavior | 25,800,000 | 0.04 | 0.14 | 32,300,000 | 0.14 | 0.07 | 76,300,000 | 0.08 | 0.80 |
| Parental monitoring of screen time | 25,800,000 | 0.04 | 0.13 | 32,300,000 | 0.14 | 0.07 | 76,400,000 | 0.08 | 0.82 |
| Limiting screen time | 25,800,000 | 0.04 | 0.13 | 32,300,000 | 0.14 | 0.07 | 76,300,000 | 0.08 | 0.81 |
| *AIC* Akaike Information Criterion. *ICC* Intraclass Correlation Coefficient. | | | | | | | | | |

| Supplementary Material 4. Associations between media parenting practices (Year 3) and video games (Years 4 and 5) in the Adolescent Brain Cognitive Development (ABCD) Study (*N* = 7407) | | | | | | |
| --- | --- | --- | --- | --- | --- | --- |
|  | Frequency of Mature Video Games | | Problematic Video Game Use | | Total Video Game Time (h/d) | |
| Media parenting practice categories | *AOR* (95% CI) | *P* | *B* (95% CI) | *P* | *B* (95% CI) | *P* |
| Parental screen time modeling | 1.04 (0.98, 1.10) | 0.213 | 0.01 (-0.01, 0.03) | 0.290 | -0.03 (-0.12, 0.06) | 0.523 |
| Mealtime screen use | **1.07 (1.03, 1.11)** | **0.001** | **0.02 (0.01, 0.04)** | **0.001** | **0.14 (0.08, 0.21)** | **<0.001** |
| Bedroom screen use | 1.04 (0.99, 1.08) | 0.112 | **-0.02 (-0.04, -0.004)** | **0.016** | -0.01 (-0.09, 0.06) | 0.699 |
| Use of screens to control behavior | **1.05 (1.00, 1.09)** | **0.029** | **0.04 (0.02, 0.05)** | **<0.001** | **0.20 (0.14, 0.27)** | **<0.001** |
| Parental monitoring of screen time | 0.98 (0.94, 1.03) | 0.510 | -0.02 (-0.03, 0.003) | 0.096 | 0.01 (-0.07, 0.09) | 0.841 |
| Limiting screen time | 0.93 (0.87, 1.00) | 0.052 | -0.003 (-0.03, 0.02) | 0.808 | **-0.20 (-0.31, -0.08)** | **0.001** |
| **Bold** indicates *P* < 0.05. All six media parenting practice categories are included in each model. Models represent the abbreviated output from the mixed-effects ordinal logistic regression model or mixed-effects generalized linear models with adjustment for Year 3 age, sex, race and ethnicity, household income, highest parental education, respective video game variable, study year, and study site. *AOR* adjusted odds ratio from mixed-effects ordinal logistic regression model, *B* coefficient from mixed-effects generalized linear model, *CI* confidence interval. | | | | | | |

Supplementary Material 5. Correlation matrix of the media parenting practices


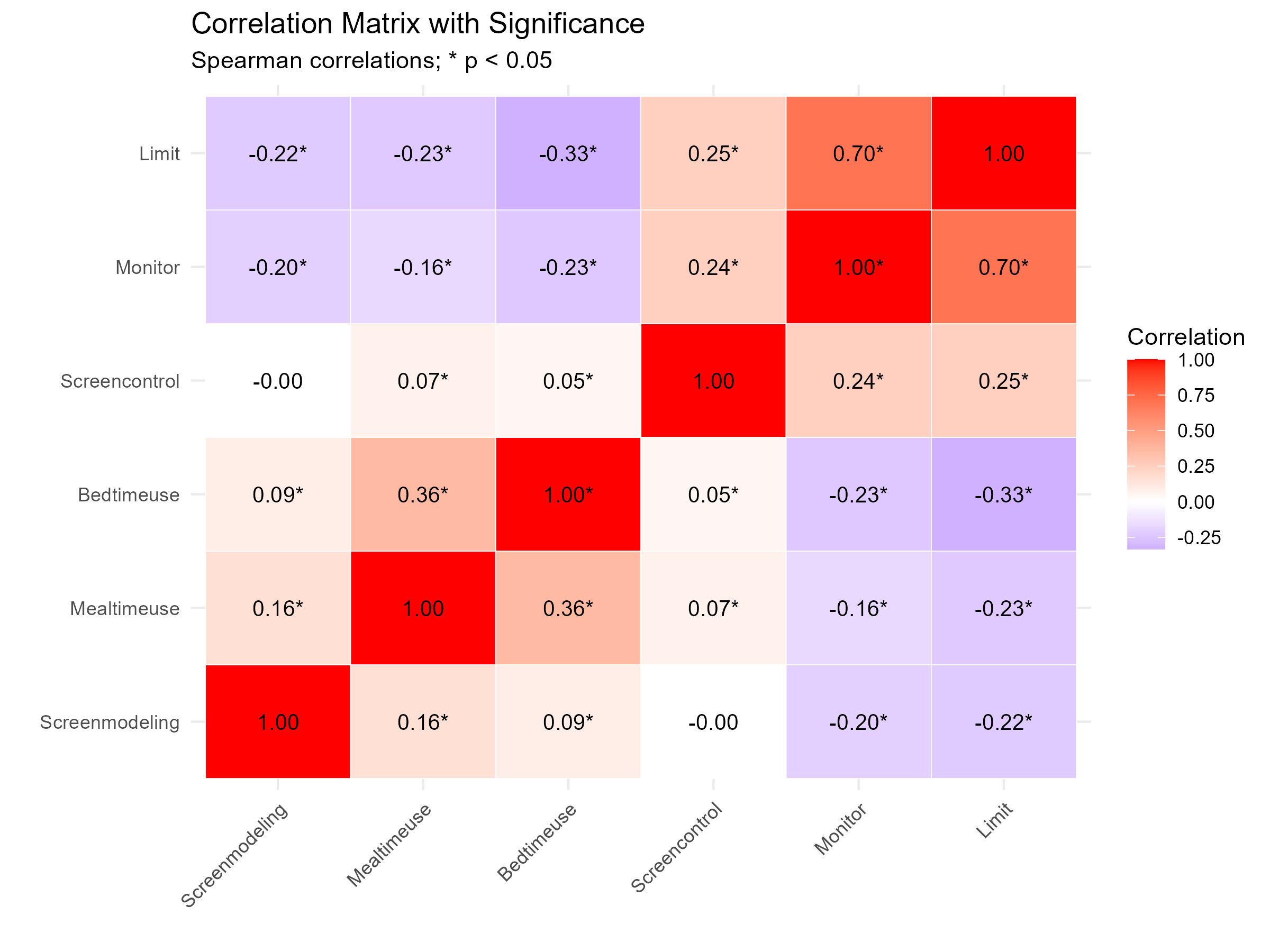

Supplement: Supplementary file 1 — Supplementary file1 (DOCX 217 KB) [file 12519_2025_1009_MOESM1_ESM.docx]
